# Purkinje Cells as Sources of Arrhythmias in Long QT Syndrome Type 3

**DOI:** 10.1038/srep13287

**Published:** 2015-08-20

**Authors:** Vivek Iyer, Danilo Roman-Campos, Kevin J. Sampson, Guoxin Kang, Glenn I. Fishman, Robert S. Kass

**Affiliations:** 1 Department of Pharmacology, Columbia University Medical Center, New York, NY; 2 Leon H. Charney Division of Cardiology, New York University School of Medicine, New York, NY.

## Abstract

Long QT syndrome (LQTS) is characterized by ventricular arrhythmias and sudden cardiac death. Purkinje cells (PC) within the specialized cardiac conduction system have unique electrophysiological properties that we hypothesize may produce the primary sources of arrhythmia in heritable LQTS. LQTS type 3 (LQT3) transgenic mice harboring the ΔKPQ^+/−^ mutation were crossed with *Contactin2-EGFP* BAC transgenic mice, which express a fluorescent reporter gene within the Purkinje fiber network. Isolated ventricular myocytes (VMs) (EGFP^−^) and PCs (EGFP^+^) from wild type and ΔKPQ mutant hearts were compared using the whole-cell patch clamp technique and microfluorimetry of calcium transients. Increased late sodium current was seen in ΔKPQ-PCs and ΔKPQ-VMs, with larger density in ΔKPQ-PCs. Marked prolongation of action potential duration of ΔKPQ-PCs was seen compared to ΔKPQ-VMs. ΔKPQ-PCs, but not ΔKPQ-VMs, exhibited frequent early afterdepolarizations, which corresponded to repetitive oscillations of intracellular calcium. Abnormalities in cell repolarization were reversed with exposure to mexiletine. We present the first direct experimental evidence that PCs are uniquely sensitive to LQT3 mutations, displaying electrophysiological behavior that is highly pro-arrhythmic.

Purkinje cells (PCs) form an extensive specialized conduction system network that ultimately interfaces with ventricular myocytes (VMs) to initiate contraction of the ventricular muscle. PC electrophysiology differs from that of neighboring VMs in several important regards, including action potential (AP) shape, AP duration (APD), and types and balance of membrane currents^1^. Computational studies predict that the cellular sources of arrhythmia such as early afterdepolarizations (EADs) may occur preferentially in PCs, owing to their unique electrophysiological features^2^,^3^. Experiments using pharmacologic manipulation of PCs suggest that they are particularly prone to EADs under a variety of conditions^4^,^5^,^6^,^7^.

The long QT syndrome (LQTS) is a family of inherited arrhythmic disorders caused by dysfunction of one of 13 different ion channel complex proteins^8^. Gain of function mutations of *SCN5A*, the gene encoding the sodium channel alpha subunit Na_v_1.5, lead to LQTS type 3 (LQT3). The most thoroughly characterized LQT3 mutation, deletion of three residues in the linker between domains III and IV of Na_v_1.5 (ΔKPQ), causes defects in the channel inactivation gate such that persistent, non-inactivating current is carried through the channel^9^. This additional inward current serves to prolong APD, promoting formation of arrhythmia. However, to date, no study has reported on whether heritable LQTS mutations, such as the ΔKPQ LQT3 mutation, are especially proarrhythmic within PCs, given their distinct electrophysiological properties compared to VMs.

In the present study, we utilize the *Cntn2-EGFP* mouse model^10^,^11^ and a well-established murine model of LQT3, the *SCN5A* ΔKPQ^+/−^ mutant mouse^12^,^13^ (characterized by ventricular arrhythmias and APD prolongation) to isolate and functionally characterize PCs and compare them to VMs. We demonstrate significant differences between mutant PCs (ΔKPQ-PCs) and VMs (ΔKPQ-VMs) with respect to cell repolarization, late sodium current, calcium cycling, and formation of EADs. Our findings provide direct evidence that cells in the Purkinje fiber network are characterized by increased susceptibility to arrhythmia formation in congenital LQTS compared to ventricular myocardium.

## Methods

### Animal model

For an expanded materials and methods section, see the online supplement. Briefly, we used the previously described *Cntn2-EGFP* transgenic mice and ΔKPQ^+/−^ mutant mice^10^,^11^,^12^,^14^. All mice studied were F1 crosses between the two strains and used at 8–12 weeks of age. Cardiomyocyte isolation and calcium imaging were carried out as described in our previous publications^10^,^11^,^14^. Animals were sacrificed by first providing isoflurane anesthesia via vaporizer, followed by cervical dislocation to assure euthanasia; all experiments were performed according to protocols approved by the NYU Institutional Animal Care and Use Committee and conformed to the National Institutes of Health (NIH) guidelines for the care and use of Laboratory Animals.

### Electrophysiology

Whole-cell voltage- and current-clamp recordings were obtained using Axopatch 200B amplifier (Axon Instruments Inc.). We recorded 10–50 APs per cell at 1 Hz, with selected experiments at 0.2 Hz as noted in the text. All sodium currents presented and used for analysis were tetrodotoxin (50 μM)-sensitive current. Single step late non-inactivated sodium current (I_NaL_) was measured at 200 ms during depolarization to −10 mV, from a holding potential of −90 mV.

### Statistical analysis

Data reported represent mean and standard error of the mean; statistical significance was determined using ANOVA (using a nested design for cells from each animal) and the Fisher’s exact test for categorical variables. In case of non-normal distribution of data (such as APD data, and the pre- and post-mexiletine APDs shown in Supplement Table 1), a non-parametric Wilcoxon rank sum test was used (Wilcoxon signed-rank test for paired data). A p value < 0.05 was considered statistically significant.

## Results

### Late sodium current in ΔKPQ-VMs versus PCs

**Figure 1:**
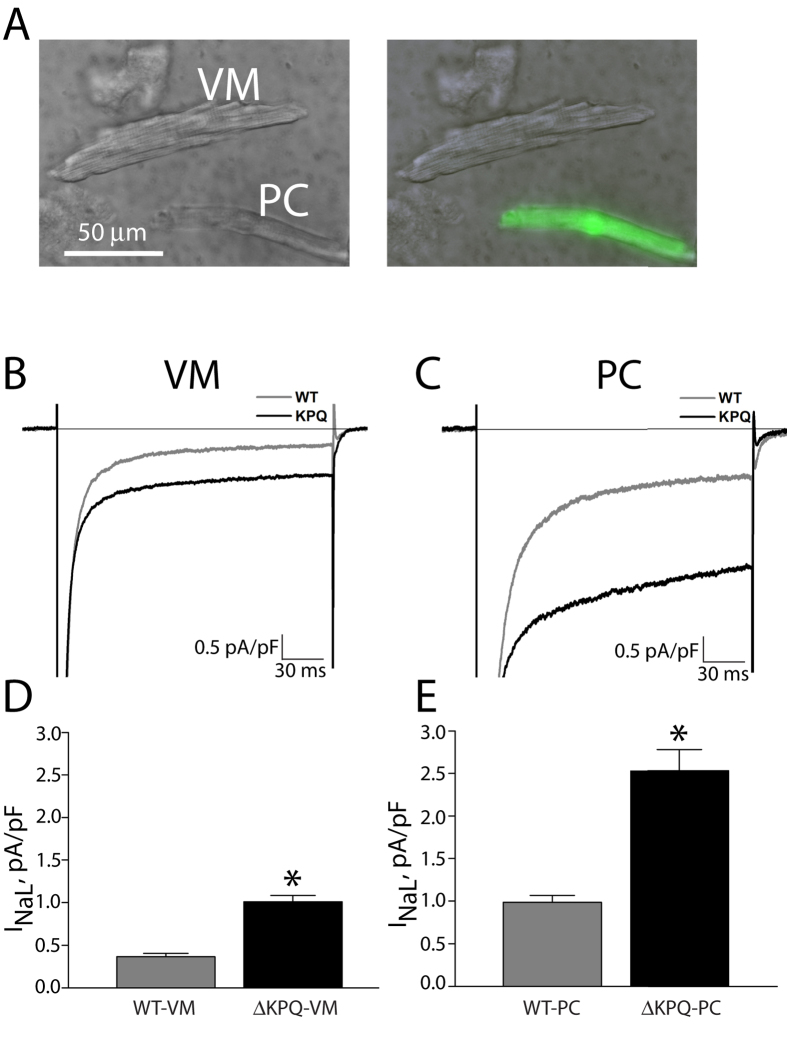
Representative cells and voltage-clamp recordings for VM and PCs. Panel (**A**) Photomicrograph of isolated PC and VM; the green fluorescent cell expresses *Cntn2-EGFP* and represents a typical PC, and the cell without fluorescence represents a typical VM. Panels (**B**,**C**) Representative whole cell currents recorded for a step pulse (200 ms at −10 mV; pulse frequency, 0.5 Hz) from VMs (panel (**B**)) and PCs (panel (**C**), peak currents are off scale). Gray trace represents a control cell, and black trace represents a ΔKPQ cell. Panels (**D**,**E**) Bar graphs summarizing absolute magnitude of I_NaL_ (I_Na_ measured at 200 ms) normalized by cell capacitance. *p < 0.05 for WT vs ΔKPQ. WT-VM: N = 15, 5 animals; ΔKPQ-VM: N = 17, 5 animals; WT-PC: N = 15, 5 animals; ΔKPQ-PC: N = 23, 5 animals. WT: wild type, VM: ventricular myocyte, PC: Purkinje cell.

Mouse PCs were easily identified via green fluorescence and morphological differences (Fig. 1A), and had lower capacitance than VMs (corresponding to lesser surface area of the longer, thinner “spindle” shape reported in PCs from mice^15^), measuring on average 53.9 ± 15.8 pF versus 136.4 ± 44.7 pF (p < 0.01).

Figure 1B,C shows I_Na_ recorded in WT cells (WT-VM and WT-PC), a typical rapidly activating and rapidly inactivating current, with a small non-inactivating persistent component. I_NaL_ for WT-VM measured 0.34 +/− 0.04 pA/pF, compared to 0.99 +/− 0.07 for WT-PC (p < 0.01, Fig. 1D,E). Larger I_NaL_ was seen in ΔKPQ cells (ΔKPQ-VM and ΔKPQ-PC). I_NaL_ for ΔKPQ-VM measured 1.00 +/− 0.07 pA/pF, compared to 2.54 +/− 0.23 in ΔKPQ-PC (p < 0.01). For both WT and ΔKPQ cells, peak sodium currents were larger in PCs than VMs, though quantitative measurement was not possible since the measured currents were often of such large amplitude that reliable voltage control was not always achieved.

### APs recorded from PC and VM have distinct properties

**Figure 2:**
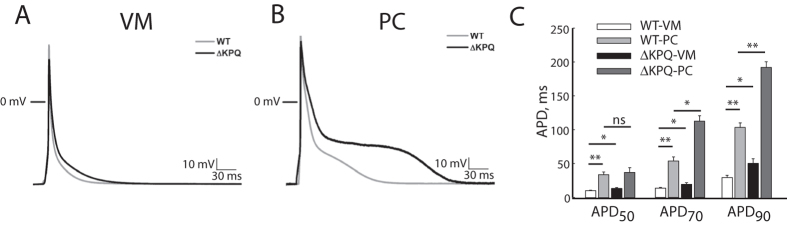
Features of action potentials recorded at 1 Hz. Panel (**A**) Representative VM action potentials. 0 mV represented by horizontal line; gray trace: WT, black trace: ΔKPQ. Panel (**B**) Representative PC action potentials. 0 mV represented by horizontal line; gray trace: WT, black trace: ΔKPQ. Panel (**C**) Bar graphs summarizing action potential duration measured at 50% of repolarization (APD_50_), 70% repolarization (APD_70_) and 90% repolarization (APD_90_). White bar: WT-VM, light gray bar: WT-PC, black bar: ΔKPQ-VM, dark gray bar: ΔKPQ-PC. *p < 0.05, **p < 0.01. WT-VM: N = 16, 6 animals; ΔKPQ-VM: N = 12, 5 animals; WT-PC: N = 16, 6 animals; ΔKPQ-PC: N = 20, 5 animals. APD: action potential duration.

APs recorded from WT-PCs were longer than those in WT-VMs, with an APD measured at 90% repolarization (APD_90_) of 104.3 +/− 6.8 ms versus 30.4 +/− 3.1 ms (p < 0.01). Representative APs are shown in Fig. 2A,B. ΔKPQ-VMs had longer APD_90_ than WT-VM (p < 0.05). ΔKPQ-PC APs were significantly longer than both WT-PC and ΔKPQ-VM APs, with an average APD_90_ (in cells that consistently repolarized) of 193.0 +/− 28.4 ms (p < 0.01 vs. WT-PC and p < 0.01 vs. ΔKPQ-VM). Group data for APDs at 50%, 70%, and 90% repolarization are compared for the different cell types in Fig. 2C.

**Figure 3:**
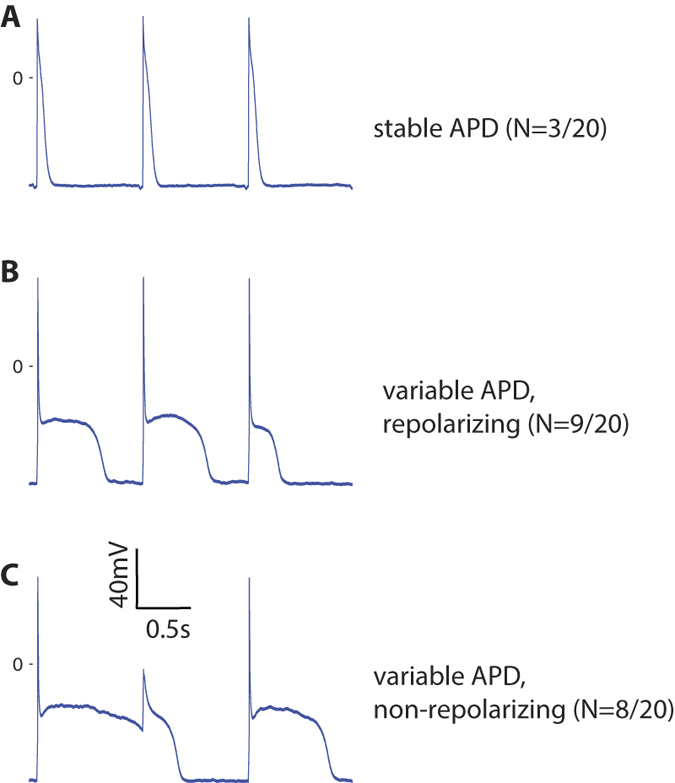
Action potentials recorded from ΔKPQ-PCs (N = 20, 5 animals) show three different behaviors. Panel (**A**) Representative action potentials with essentially stable morphology and duration (N = 3 cells demonstrated this behavior). Panel (**B**) Representative action potentials with marked variation of morphology and duration from beat to beat (N = 9 cells demonstrated this behavior). Panel (**C**) Example action potentials with intermittent failure of repolarization before arrival of a subsequent stimulus (N = 8 cells demonstrated this behavior). APD: action potential duration.

Only a minority of ΔKPQ-PCs showed stable AP morphology from sweep to sweep (Fig. 3A for representative stable APs). Instead, the characteristic behavior of ΔKPQ-PCs was profound beat-to-beat variability in AP duration and morphology (Fig. 3B). At least 10 consecutive sweeps per cell (and as many as 50, if the cell showed stable recordings) were used for each cell analyzed. We identified all cells that showed >50 ms difference in APD_90_ between the longest and shortest APs recorded during these sweeps. This degree of variation occurred in 17/20 ΔKPQ-PCs, compared with 0/12 ΔKPQ-VMs, 3/16 WT-PCs, and 0/16 WT-VMs (p < 0.01 for ΔKPQ-PC vs. all other groups; p = NS for WT-PC vs. both VM groups). In many cells, ΔKPQ-PCs intermittently failed to completely repolarize before the next stimulus, indicating an APD of at least 1000 ms (note second stimulus in Fig. 3C occurs during the extended plateau phase of first AP). This failure to repolarize occurred in 8/20 ΔKPQ-PCs, and was not seen in any other cell type.

**Figure 4:**
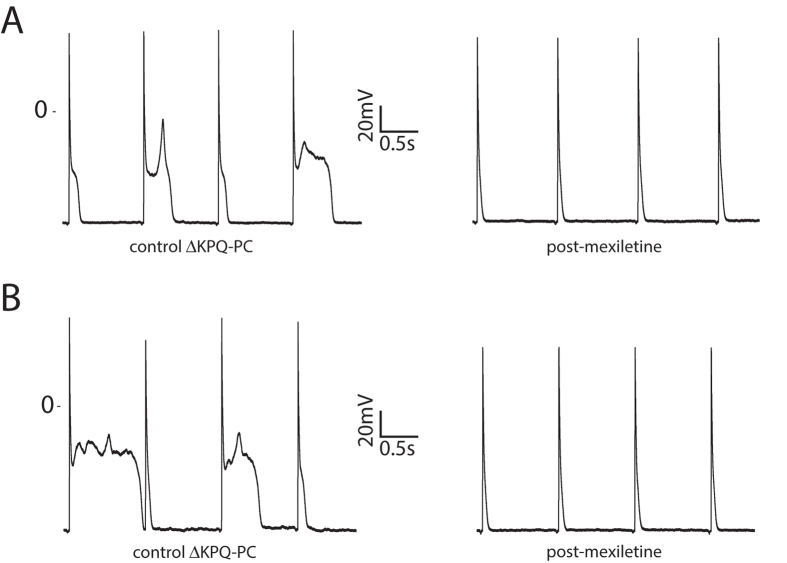
Response of △KPQ-PCs to mexiletine for a cell with wide variability of action potential morphology and duration (panel (**A**)), and a cell with near failure to repolarize (panel (**B**)). Data shown representative of N = 8 cells from 3 animals, as summarized in text.

Given the increased I_NaL_, marked APD prolongation, beat-to-beat variability of repolarization, and intermittent failure to repolarize in ΔKPQ-PCs, we analyzed the response of these cells to 20 μM mexiletine. Figure 4 shows the response of ΔKPQ-PC APs to mexiletine at 1 Hz, revealing marked shortening of APD_90_ by 149.4 +/− 42.5 ms (p < 0.01, values in Supplement Table). Indeed, post-mexiletine ΔKPQ-PC APD_90_ was not different from WT-PC values (p = NS for comparison). Mexiletine also almost completely eliminated APD variability in ΔKPQ-PCs (8/8 cells with at least 50 ms APD variability before mexiletine, 1/8 cells post-mexiletine, p < 0.01). As expected, mexiletine also reduced I_NaL_ at 1 Hz in ΔKPQ-PCs by 66% (−2.12 +/− 0.20 pA/pF to −0.72 +/− 0.10 pA/pF) and in ΔKPQ-VMs by 66% (−0.79 +/− 0.11 pA/pF to −0.27 +/− 0.04 pA/pF).

### EADs and intracellular calcium cycling

Severe AP prolongation in ΔKPQ-PCs was associated with formation of multiple EADs (as seen in the control APs in Fig. 4). Since we hypothesized that these EADs would be associated with disordered calcium handling, we performed imaging of intracellular calcium transients using the indo-1 fluorophore. Experiments were conducted at a slower pacing frequency of 0.2 Hz, to replicate pause-related AP prolongation and the known exacerbation of phenotype with bradycardia in LQT3, and to allow for complete repolarization and characterization of APs.

**Figure 5:**
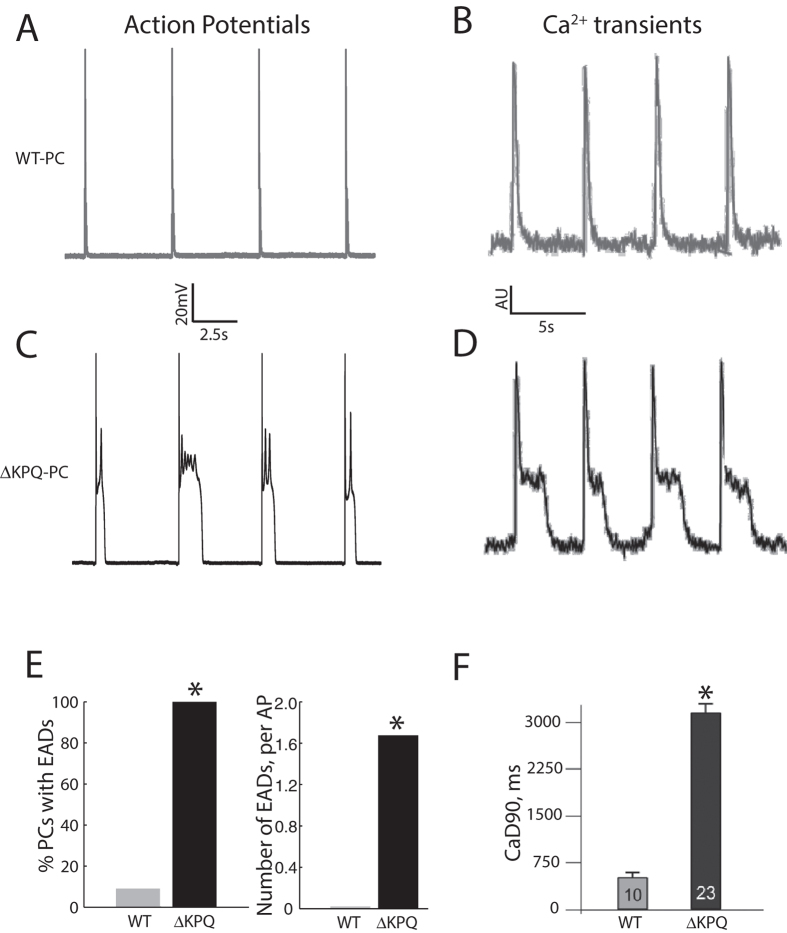
PCs carrying the ΔKPQ mutation show early afterdepolarizations associated with disordered intracellular calcium cycling. Panel (**A**,**B**) Consecutive WT-PC action potentials (Panel (**A**)) and a representative calcium transient (Panel (**B**)). Panel (**C**,**D**) Consecutive action potentials from a ΔKPQ-PC (Panel (**C**)) and a representative calcium transient (Panel (**D**)) show prolonged duration, with repetitive plateau depolarizations and oscillation in calcium transient. Panel (**E**) Summary data showing number of cells with early afterdepolarizations and number of early afterdepolarizations for ΔKPQ-PCs compared to WT-PCs. Panel (**F**) Summary data showing the duration of calcium transients as assessed by time-interval to 90% Ca^2+^ decay (CaD90) for ΔKPQ-PCs compared to WT-PCs. *p < 0.05. For action potential experiments (panel (**E**)), WT-PC: N = 11, 3 animals; ΔKPQ-PC: N = 7, 3 animals. For calcium imaging experiments (panel (**F**)), WT-PC: N = 10, 4 animals; ΔKPQ-PC: N = 23, 5 animals. WT: wild type, CaD90: time-interval to 90% Ca^2+^ decay, EAD: early afterdepolarization.

Representative PC APs with EADs (ΔKPQ cell) and without EADs (WT cell) are shown in Fig. 5 (panels A and C) alongside representative intracellular calcium transients (panels B and D). In ΔKPQ-PCs, 7/7 cells exhibited at least one EAD during the drive train, compared to 0/8 ΔKPQ-VMs, 1/11 WT-PCs and 0/8 WT-VMs (p < 0.05 for ΔKPQ-PC vs. each group). In total, 114 EADs were present in ΔKPQ-PCs (N = 68 sweeps, representing on average 1.7 EADs per AP), with some sweeps showing sequential EADs during a single AP plateau (as summarized in Fig. 5E). Calcium transients in WT-PCs were of relatively short duration, without a substantial plateau phase. The long APs in ΔKPQ-PCs were associated with oscillations in the calcium transient. Corresponding to the multiple EAD events, calcium transient duration was prolonged in KPQ-PCs by nearly an order of magnitude (Fig. 5F).

## Discussion

In this study, we provide the first direct experimental evidence that PCs are highly prone to repolarization abnormalities in the context of a canonical LQTS mutation, and may serve as the sources of arrhythmia. We specifically demonstrate that 1) murine PCs (WT and ΔKPQ) show a larger absolute I_NaL_ compared to their VM counterparts, 2) ΔKPQ-PCs manifest profound beat-to-beat variability and frequent large amplitude EADs, behavior not seen in VMs carrying the mutation, 3) abnormalities in repolarization in mutant PCs are reversed with I_NaL_ blockade, and 4) disordered intracellular calcium cycling accompanies the EADs in mutant PCs. Our findings establish that both cell type and altered channel biophysics may contribute to determine the severity of phenotype in congenital LQTS.

### Impact of ΔKPQ on PC electrophysiology

The APs recorded in WT-PCs and WT-VMs compare favorably to previously reported results^15^. PC APs have rapid early repolarization and a brief plateau potential at a relatively hyperpolarized potential (−60 mV). In contrast, few VM APs show any plateau potential, instead smoothly returning to resting potential, with a shorter APD. While I_NaL_ has not been previously measured in PCs carrying the ΔKPQ mutation, the currents we measured in ΔKPQ-PCs qualitatively resemble prior recordings of ΔKPQ I_Na_^12^, with rapid initial inactivation and persistent, plateau-like non-inactivating current. The more dramatic APD prolongation observed in ΔKPQ-PCs demonstrates that the PC AP is particularly sensitive to additional I_NaL_.

### EADs in PCs and not VMs

Our ΔKPQ-PCs are characterized by marked lengthening of the plateau phase compared with VM cells, as well as frequent, and often repetitive EADs. A previous study recorded monophasic action potentials from mouse hearts carrying the ΔKPQ mutation, showing prolonged repolarization with a plateau phase^12^; however, when recording monophasic action potentials in intact heart, APs from PCs and VMs cannot be studied independently. In the present work, we study abnormalities separately in ΔKPQ-VMs and ΔKPQ-PCs, observing that a more severe phenotype is seen in PCs. In experiments with imaging of calcium transients (Fig. 5), we demonstrate oscillations in plateau calcium, associated with EAD formation, which occurred in PCs alone. In a large animal Purkinje fiber preparation, January and colleagues demonstrated that EADs arise from reactivation of calcium currents during a prolonged plateau phase^16^. The sustained plateau phase characteristic of ΔKPQ-PC APs can be expected to promote such a mechanism, leading to repetitive calcium-dependent formation of EADs. Further, the EADs we have demonstrated are of magnitude large enough to plausibly contribute to arrhythmic activity.

### Mechanistic basis for abnormal repolarization in PCs

The larger absolute I_NaL_ is likely the most important factor in producing repolarization abnormalities in ΔKPQ-PCs, since targeted inhibition of I_NaL_ with mexiletine readily abolishes EADs and normalizes repolarization in our preparation. However, we cannot exclude the possibility that the diversity of depolarizing and repolarizing currents between PCs and VMs contributes to the preferential formation of EADs in PCs. PCs are known to express T-type calcium current unlike VMs, and have different densities of repolarizing potassium currents^15^. There are also known differences in expression of sodium channel isoforms between PCs and VMs^1^,^17^. It is unlikely that differences in the magnitude of these membrane currents can solely account for the emergence of EADs in one cell type and not the other^15^, but they may serve a modulating role in determining APD.

### Beat-to-beat variability of APs in ΔKPQ-PCs

In most ΔKPQ-PCs, we observed marked beat-to-beat variability in AP duration and morphology. In the intact heart, this could establish heterogeneity of refractoriness across the Purkinje network, as well as between PC and VM cells, conditions that are favorable for initiation of reentrant arrhythmias^5^. While electrotonic interactions between cells may reduce the extent of this heterogeneity, the substrate is nevertheless likely to be highly arrhythmogenic. In future work, whole heart computational models will be implemented to investigate the factors that lead to arrhythmia propagation at these PC-VM junctions^18^.

### Use of transgenic murine models in the study of LQTS

The ability to study mutations within different native cellular environments is a powerful strategy to uncover arrhythmogenic mechanisms and identify potential therapeutic targets. Unlike experiments using heterologous expression systems, in the present work mutant channels are studied with a normal complement of accessory subunits, chaperones and regulatory proteins that modulate channel function^19^. Such studies may identify individual cell types such as PCs that are uniquely sensitive to specific disease-causing mutations, as we demonstrate for the first time here for ΔKPQ and LQT3.

### Limitations

In this study, we performed our experiments at room temperature at pacing frequencies designed to approximate human heart rates, with selected experiments at a slower pacing rate, conditions which match our previous work studying this mutation^13^. Though the pacing rates differ from the native mouse heart rate (and indeed, other important differences exist between mouse and human cellular electrophysiology), this frequency is also more relevant for assessment of the mechanism of LQT3-related arrhythmias, which form more commonly during prevailing bradycardia^20^.

As with any drug, mexiletine can have off-channel effects, particularly at high doses^21^, which could in theory indirectly contribute to the normalization of APD and elimination of EADs in ΔKPQ-PCs. However, at the lower 20 μM dose used in this study, the predominant effect is on I_Na_. At this dose I_NaL_ is substantially reduced in all cell types. We therefore believe that our post-drug results reflect the effects of I_NaL_ inhibition.

Finally, we focused on single cell electrophysiological differences between PCs and VMs, and did not attempt to study propagation of these events in a whole heart preparation. As described above, we plan to study this using a computational approach, but additional experimental characterization must first be performed to determine the nature and number of interface points between the PC network and VMs^22^. These experiments and accompanying simulations are underway but outside the scope of the current study.

## Additional Information

**How to cite this article**: Iyer, V. *et al.* Purkinje Cells as Sources of Arrhythmias in Long QT Syndrome Type 3. *Sci. Rep.* **5**, 13287; doi: 10.1038/srep13287 (2015).

## Supplementary Material

Supplementary Information
