# Supplementary material for: Purkinje Cells as Sources of Arrhythmias in Long QT Syndrome Type 3
Source: Sci Rep. 2015 Aug 20;5:13287. doi: 10.1038/srep13287 (PMC4542521; doi:10.1038/srep13287)
Supplement: Supplementary Information [file srep13287-s1.doc]

**Supplementary Information**

**Purkinje Cells as Sources of Arrhythmias in Long QT Syndrome Type 3**

Vivek Iyer*^+^, Danilo Roman-Campos*^+^, Kevin J. Sampson^+^, Guoxin Kang^&^; Glenn I. Fishman^&^, Robert S. Kass^+^

^+^ Department of Pharmacology, Columbia University Medical Center, New York, NY

^&^ Leon H. Charney Division of Cardiology, New York University School of Medicine, New York, NY

**Corresponding authors**

Robert S. Kass PhD

Department of Pharmacology, College of Physicians & Surgeons of Columbia University

630 W 168th St, PH 7W 318, New York, NY 10032
Fax: 212-305-3545, Telephone: 212-305-3720

E-mail: rsk20@columbia.edu

Glenn I. Fishman MD

Leon H. Charney Division of Cardiology

New York University School of Medicine

522 First Ave, Smilow 801, New York, NY 10016

Fax: 212-263-3972, Telephone: 212-263-3967

E-mail: glenn.fishman@nyumc.org

*Dr. Iyer and Dr. Roman-Campos contributed equally to this work.

| **Cell type** | **APD_90_**  **pre-drug, ms** | **ΔAPD_90_**  **post-drug, ms** | **p-value** | **dV/dt max,**  **V/s** | **ΔTime to peak post-drug, ms** | **p-value** |
| --- | --- | --- | --- | --- | --- | --- |
| **WT-VM** | 27.0 +/- 5.7 | 1.6 +/- 0.6 | *p=0.037* | 193.3 +/- 19.7 | 1.4 +/- 0.2 | *p=0.002* |
| **WT-PC** | 145.7 +/- 43.7 | -61.5 +/-35.7 | *p=0.008* | 268.8+/-19.7 | 1.0 +/- 0.1 | *p=0.004* |
| **ΔKPQ-VM** | 43.5 +/- 5.9 | 1.8 +/- 2.5 | *p=0.69* | 159.5 +/-16.4 | 1.8 +/- 0.3 | *p=0.016* |
| **ΔKPQ-PC** | 252.0 +/- 42.8 | -149.4 +/- 42.5 | *p=0.008* | 168.2 +/- 12.5 | 2.4 +/- 0.9 | *p=0.008* |

Supplement Table. Features of action potentials and effect of mexiletine in the four cell types. In VMs, there was minimal prolongation of action potential duration (APD_90_, which was statistically significant in WT cells), which is partially accounted by delay to reaching peak of action potential in the presence of drug (right side of table, peak dV/dt pre-drug is also provided for reference). In PCs, there was marked statistically significant reduction in APD_90_ post-drug.

**Detailed Methods**

### Mutant Mice

Animals were sacrificed by first providing isoflurane anesthesia via vaporizer, followed by cervical dislocation to assure euthanasia; all experiments were performed according to protocols approved by the NYU Institutional Animal Care and Use Committee and conformed to the National Institutes of Health (NIH) guidelines for the care and use of Laboratory Animals. Cntn2-EGFP BAC transgenic mice and ΔKPQ^+/-^ mutant mice have both been previously described^1-3^. All mice studied were F1 crosses between the two strains and used at 8-12 weeks of age. To minimize experimental variability, data from ventricular myocytes (VMs) and Purkinje cells (PCs) from the same preparation were always collected on the same day.

**Cardiomyocyte isolation**

Hearts were quickly removed, mounted and perfused using Langendorff apparatus for 5 min with nominally Ca^2+^-free solution containing (in mM): 113 NaCl, 4.7 KCl, 0.6 NaH_2_PO_4_, 0.6 KH_2_PO_4_, 1.2 MgSO_4_.7H_2_O, NaHCO_3_, KHCO_3_, 10 HEPES, 30 Taurine, 5.5 Glucose. Next, hearts were perfused for 10–15 min at 37 °C with a solution containing 1 mg/ml collagenase (CLS-2, Worthington Biochemical, Lakewood, NJ, USA), 0.06 mg/ml trypsin (T- 8003, Sigma, St. Louis, MO, USA), and 0.06 mg/ml protease (P-5147, Sigma). The digested heart was removed from the cannula, and supraventricular cells were discharged and the remaining tissue was separated, minced, gently agitated, centrifuged (1000 rpm, 10 s, room temperature) and stored in the same solution used for isolation added 0.5 μM of CaCl_2_. The isolated cardiac cells were stored at room temperature and used within 6–8 h of isolation. Only calcium-tolerant, quiescent, rod-shaped cardiac cells were studied.

**Ca2+ imaging**

Ca2+ imaging was carried out as described previously^3^. Briefly, cells were exposed to a dye loading solution consisting of a standard Tyrode’s solution containing (in mm): 140 NaCl, 4 KCl, 2 CaCl2, 1 MgCl2, 10 HEPES, and 5.6 glucose. The solution was supplemented with 2.5 μm x-Rhod-1 acetoxymethyl ester (x-Rhod-1/AM; Invitrogen Inc., Eugene, OR). Cells were exposed to x-Rhod-1/AM for 6 min at 22°C. The loading solution was removed, and cells were washed and equilibrated in fresh Tyrode’s solution for 30 min at 22°C to allow de-esterification of the dye before recording. Epifluorescence imaging was used to identify the GFP-expressing and non-expressing cells. Fluorescent signals were acquired using a 40X UVF objective (numerical aperture 1.0, Nikon), and single excitation wavelength microfluorimetry was performed using a photomultiplier tube system (IonOptix, Milton, MA). Cells were field-stimulated at 0.2Hz to achieve steady-state. Freshly isolated cells were incubated with 2μM Indo-1/AM (Invitrogen) at room temperature for 15min. Then the loading solution was replaced with fresh Tyrode’s solution for 30 min to allow deesterification of the dye before recording. The Indo-1 loaded cells were illuminated by a Xenon lamp (excitation was 340nm) and the dual emission light (405-nm and 495-nm) was collected by two separate PMT tubes. The fluorescence ratio was calculated by Ionwizard (IonOptix, Milton, MA).

**Whole-cell ruptured patch-clamp technique**

Whole-cell voltage- and current-clamp recordings were obtained using Axopatch 200B amplifier (Axon Instruments Inc.). Upon attaining break-in, 2 to 4 minutes were allowed to pass for the pipette solution to equilibrate with the cellular interior. The experiments were carried out at room temperature (25 °C). The recording electrodes had resistances from 0.5 to 1.5 MΩ. Current recordings were low-pass filtered (2 kHz) and digitized at 10 or 50 kHz before being stored on a computer. Cells showing series resistance larger than 5 MΩ were not used in the analysis. Series resistance was compensated digitally during voltage-clamp experiments at 40 to 70 %.

To measure action potentials, the pipette solution contained (in mM): 150 KCl, 10 HEPES, 1 MgCl2, 5.3 CaCl2, 10 EGTA, ATP-Na, pH set to 7.3 with KOH. We used Tyrode's as bath solution (in mM) 140 NaCl, 5.4 KCl, 0.5 MgCl2, 1.8 CaCl2, 0.33 NaH2PO4 , 5 HEPES, 11 glucose, with pH set at 7.4 with NaOH. Cells were stimulated in current clamp mode with rectangular (3-7 ms) depolarizing current pulses with supra-threshold current. We recorded 10–50 action potentials per cell at 1 Hz, with selected experiments at 0.2 Hz to replicate pause protocols.

To record macroscopic sodium currents we used the following internal solution (in mM): 50 aspartic acid, 60 CsCl, 5 Na2ATP, 11 EGTA, 10 HEPES, 4.27 CaCl2 (resulting in a final [Ca2+]_i_ of 100nM), and 1 MgCl2, pH 7.4 adjusted with CsOH. The external solutions for sodium current experiments contained (in mM): 130 NaCl, 2 CaCl2, 5 CsCl, 1.2 MgCl2, 10 HEPES, and 5 glucose, with pH 7.4 adjusted with NaOH. 1 uM isradipine and 10 uM nifedipine were added on the day of the experiment. All sodium currents presented and used for analysis are tetrodotoxin (50μM)-sensitive current.

Single step late non-inactivated sodium current (INaL) was measured as the tetrodotoxin-sensitive current measured at 200 ms during depolarization to -10 mV, from a holding potential of -90 mV, applied every 2 seconds.

**Data Analysis**

PClamp10.2 (Axon Instruments) was used for data acquisition and initial analysis. Analysis was carried out in Origin 7.0 (Microcal Software, Northampton, MA) and in MATLAB (The Mathworks, Natick, MA).

Data reported represent mean and standard error of the mean; statistical significance was determined using ANOVA (using a nested design for cells from each animal) and the Fisher’s exact test for categorical variables. In case of skew distribution of data (such as APD, for the data in Figure 2, and the pre- and post- mexiletine APDs shown in Supplement Table 1), the assumptions inherent to ANOVA are not met. For these data, a non-parametric Wilcoxon rank sum test was used, or Wilcoxon signed-rank test for paired data. A p value of < 0.05 was considered statistically significant.

**Supplement references**

1 Kang, G. *et al.* Purkinje cells from RyR2 mutant mice are highly arrhythmogenic but responsive to targeted therapy. *Circulation research* **107**, 512-519, doi:10.1161/CIRCRESAHA.110.221481 (2010).

2 Nuyens, D. *et al.* Abrupt rate accelerations or premature beats cause life-threatening arrhythmias in mice with long-QT3 syndrome. *Nature medicine* **7**, 1021-1027, doi:10.1038/nm0901-1021 (2001).

3 Pallante, B. A. *et al.* Contactin-2 expression in the cardiac Purkinje fiber network. *Circulation. Arrhythmia and electrophysiology* **3**, 186-194, doi:10.1161/CIRCEP.109.928820 (2010).
